# Transform-Based Multiresolution Decomposition for Degradation Detection in Cellular Networks

**DOI:** 10.3390/s20195645

**Published:** 2020-10-02

**Authors:** Sergio Fortes, Pablo Muñoz, Inmaculada Serrano, Raquel Barco

**Affiliations:** 1 Departamento de Ingeniería de Comunicaciones, Campus de Teatinos s/n, Andalucía Tech, Universidad de Málaga, 29071 Málaga, Spain; rbm@ic.uma.es; 2 Department of Signal Theory, Telematics and Communications (TSTC), Universidad de Granada, 18071 Granada, Spain; pabloml@ugr.es; 3 Ericsson, 29590 Málaga, Spain; inmaculada.serrano@ericsson.com

**Keywords:** cellular management, failure detection, self-healing, transform-based, wavelet

## Abstract

Anomaly detection in the performance of the huge number of elements that are part of cellular networks (base stations, core entities, and user equipment) is one of the most time consuming and key activities for supporting failure management procedures and ensuring the required performance of the telecommunication services. This activity originally relied on direct human inspection of cellular metrics (counters, key performance indicators, etc.). Currently, degradation detection procedures have experienced an evolution towards the use of automatic mechanisms of statistical analysis and machine learning. However, pre-existent solutions typically rely on the manual definition of the values to be considered abnormal or on large sets of labeled data, highly reducing their performance in the presence of long-term trends in the metrics or previously unknown patterns of degradation. In this field, the present work proposes a novel application of transform-based analysis, using wavelet transform, for the detection and study of network degradations. The proposed system is tested using cell-level metrics obtained from a real-world LTE cellular network, showing its capabilities to detect and characterize anomalies of different patterns and in the presence of varied temporal trends. This is performed without the need for manually establishing normality thresholds and taking advantage of wavelet transform capabilities to separate the metrics in multiple time-frequency components. Our results show how direct statistical analysis of these components allows for a successful detection of anomalies beyond the capabilities of detection of previous methods.

## 1. Introduction

The complexity of cellular networks is continuously growing. This complexity increases the costs of the network infrastructure and those of its operation, administration, and management (OAM) activities. The huge number of indicators, counters, alarms, and configuration parameters transform network monitoring into a complicated task.

In this field, the concept of self-healing, as part of the self-organizing network (SON) paradigm [1,2], aims to automate the tasks associated with network failure management, achieving a more reliable service provision with minimum operational costs. Self-healing includes the tasks of the detection of degradations in the network service (familiarly known also as problems), diagnosis of the root cause or fault generating the problem, compensation of the degradation, and the recovery of the system to its original state.

In this scheme, the detection of network problems is one essential task for the posterior failure management activities. The fast and reliable detection of failures is of paramount importance in current cellular infrastructure, especially given its increasing heterogeneity due to the coexistence of multiple technologies (GSM, UMTS, LTE) and deployment models (macrocell, small cell). Here, an effective analysis of cellular metrics coming from the different elements of the network (terminals, base stations, sectors, etc.) is essential for failure management.

In cellular networks, failure detection is based on the analysis of network metrics (indicators, key performance indicators (KPIs), counters, alarms, and mobile traces) by means of identifying abnormal/unhealthy values in its temporal series. Classic techniques for cell degradation detection are based on the definition of threshold values (upper or lower) [3,4,5]. If the values for a given metric violate such thresholds, it is considered degraded. Threshold definitions are typically dependent on human knowledge about the correct values of the metrics [6]. This is a main drawback for such mechanisms as they require expertise that is not available in many cases. Even when that knowledge is available, the process is very time consuming, costly, and unreliable due to the large number of indicators and the different conditions of distinct network elements [6]. In addition, with fixed threshold schemes, it is not possible to adapt to the daily, weekly, and long-term variations of the traffic load. Alternatively, machine learning (ML)-based approaches can be used to automatically establish the detection thresholds or classification models. However, such approaches require large labeled datasets [7], also very rarely available in real networks [6]. Furthermore, such training data might not be applicable to different networks or cells from the ones where it was gathered.

Our hypothesis is that transform-based decomposition can help to overcome the limitations of previous approaches. Transforms consist of functions that map a particular metric into a space that simplifies its analysis. Typical transforms include Fourier [8] and wavelet solutions [9]. These mechanisms can be applied to properly decompose the different trends present a in time-series, and they have been used for the detection of abnormal behavior in other fields, such as phonocardiograms [10].

However, there is an important knowledge gap in the application of transform-based approaches and, particularly, wavelet transforms, in cellular networks, where to our knowledge, they have not been applied for metric-based anomaly detection [7,11]. Moreover, the application of such techniques is not straightforward, due to the particularities of cellular metrics, which include a wide variety of metrics showing different resolutions (e.g., hourly, daily), pseudoperiodic trends (e.g., daily, weekly, monthly), and long-term variations (e.g., increasing or decreasing of the telecommunication service in an area), which will impact the metrics in a very heterogeneous manner, even for those coming from the same network element (e.g., the throughput and the number of handover failures of the same cell might present very different temporal patterns).

In order to overcome these challenges, our work (whose developments have been partially subject to a patent application [12]) proposes a framework for the detection of the degradations in a metric. This framework revolves around the multiresolution decomposition of the metric (focused on the use of discrete wavelet transform (DWT)) at different times and scales, where the scale is related to the frequency of the component. To do so, our proposed system defines a series of stages able to characterize and configure the transform decomposition in an automatic manner suitable for heterogeneous cell metrics.

The decomposition is proposed as a data transformation step able to support the following classification stage dedicated to detecting anomalies. The subdivision of the original metric into separate time-scale components containing its different temporal trends and patterns should benefit the detection, allowing for the application of simpler inference methods with improved results. In particular, a simple statistical-based outlier identification technique is implemented to show the capabilities of the proposed framework to facilitate the detection process. To demonstrate this, the complete framework is evaluated based on data coming from a real commercial cellular network, allowing for a proper detection of different degradation patterns even without labeled data or large datasets.

In this way, the present paper is organized as follows. Section 2 analyzes the related works in the field. Section 3 firstly presents the general introduction to the discrete wavelet transform, elaborating on its applicability to the objectives of the work. Secondly, it describes the proposed system for the application of these transforms in the detection of cellular metric network abnormal values, establishing the inputs, outputs, and general structure. The proposed scheme is evaluated in Section 4 based on real-life cellular metrics. Finally, Section 5 presents the main conclusions and outlook for the work.

## 2. Related Work

There is an abundant literature of related works in the field of anomaly detection for cellular networks. Where extensive surveys in this field can be found in the literature [7,11], this section will provide a summary of key references in the field. These will be summarized in Table 1. Without intending to cover all possible approaches, a set of main categories is identified for the analyzed references, mainly on the basis used for degradation identification (human knowledge, statistical analysis, pattern comparison, predictor mechanisms). In the table, the applied algorithmic techniques are identified, and a summary of the common methodology for each category is provided.

In this field, classical approaches have considered manually defined thresholds for anomaly detection. A category of works focuses on how to apply these defined thresholds or to facilitate their calculation from human knowledge. Khatib et al. [13] evolved on this by proposing a finite-state machine for the identification of degradation intervals, where the transition between states is based on the crossings of manually defined thresholds by selected KPIs. Some recent works go beyond this approach, putting the focus on the knowledge acquisition procedures to help acquire the expertise from telecommunication operator personnel. Here, Reference [14] complemented the work in [13] by applying entropy minimization discretization for the acquisition of the numerical thresholds based on a set of manually solved cases.

Other works have tried to automate the thresholding process based on the statistical analysis of the metrics. In this field, Novaczki et al. [3] established a framework for detecting degradation based on deviations from the average KPI values obtained during an observation window. From that, the work in [15] improved the previous solution by adding the analysis of the statistical profiles of the indicators; this means the probability distribution functions of each metric depending on the status of or failure in the network. In a similar fashion, Reference [16] defined a framework for the detection of outages based on the estimation of the statistical profile of the measurements being gathered by a UE in comparison with previous ones for the same location. Additionally, Muñoz et al. [17] proposed an automatic thresholding method using the metric statistics and the application of percentile-based discretization.

Where these methods allow for an automatic definition of the normality values or the classification rules, they are highly affected by the variability of the network conditions and long-term trends, which will reduce their capability to detect anomalies.

Machine learning classifier techniques, such as naive Bayesian classifiers [4], k-nearest neighbor, or support vector machine [18], can also be implemented in a binary way to identify values as degraded or not. Where these techniques automatically integrate the detection decision, their need for labeled cases and typically their analysis of the metric values in an atemporal manner can make them often underperform.

As included in Table 1, a third category of works is identified as those that revolve around the comparison of a time-series of the metric under analysis with other series with certain patterns. In this field, Cheung et al. [19] presented a technique for cell automatic failure detection based on the time-series analysis of traffic data. This was based on the search for abnormal trends in the indicators, by comparing them with baseline learned profiles of their own cell with the simultaneous traffic of neighboring cells. Furthermore, correlation-based mechanisms have been considered applicable to this problem. In [20], the degradation was detected by the correlation between the indicators of an observed cell and a neighboring cell. In particular, this occurs when the correlation coefficient between the observed cell indicator and the neighboring healthy one drops below a certain threshold. The work in [21] made use of pattern clustering for the identification of a set of metric values with predetermined fault categories that were determined with the help of expert knowledge. Muñoz et al. [22] further evolved the category of pattern comparison approaches by the use of degradation patterns, constructed based on normal time-series and synthetic degradations (e.g., impulse, ramps). Alternatively, the correlation with other external “contextual” sources, such as information on social events (e.g., sport matches, concerts, etc.) can also help to establish the presence and cause of performance anomalies in the network [23].

These pattern comparison methods typically allow coping with the presence of temporal trends in the metrics, and they do not rely on a direct definition of detection thresholds. However, for the methods using the analysis of neighboring cells’ data [19,20], these imply the existence of a close association between the compared cells’ traffic, which is not commonly the case. They also require human expertise for the identification of the patterns (or cells) to be considered normal.

Another category of solutions is the one based on prediction mechanisms. These approaches focus on the generation of a predictive model based on a set of data considered normal, generally at the initial values of the time-series under analysis. The error between the predicted values and the measured ones is used as an indicator/score of degradation. The specific threshold to consider each sample as degraded or not is typically established manually or automatically based on the general statistical distribution of such an error. Here, many different predictive models can be used, such as the AutoRegressive Integrated Moving Average (ARIMA) [18], forward linear predictors [24], or Long Short-Term Memory (LSTM) recurrent neural networks [25]. These methods, especially those using more advanced predictors, will typically require large datasets, and their performance for anomaly detection will be highly impacted by the proper configuration of the model and the variability and stationarity of the analyzed metric.

In this way, the detection of network issues remains to be fully solved in the challenging scenario caused by the variability of service behavior over days and weeks, as well as the presence of long-term variations in the use of each particular cellular element. In this field, some solutions try to simply remove the periodic or long-term components in the analysis of cell indicators [6]. However, those approaches only work in the presence of simple and predictable patterns. They also affect the information associated with trends that can be key for the network analysis.

Conversely, in order to support anomaly detection mechanisms able to analyze multiple metric behaviors, our proposal aims at the application of transform-based decomposition, particularly by wavelet transforms [9]. In this respect, there is a very extended literature in the field of transform-based data decomposition in the context of anomaly detection. From this, key related works were identified as summarized in Table 2.

Transforms have been used in the past for the detection of abnormal behavior in other fields. For example, the work in [10] made use of STFT and the Continuous Wavelet Transform (CWT) for the analysis and detection of abnormalities in phonocardiogram signals. Reference [26] applied a static threshold to wavelet transform components for the analysis of non-cellular network layer measurements (e.g., HTTP packet sizes). Furthermore, in the analysis of packet lengths with the objective of anomaly detection for network layer traces, Du et al. [27] applied DWT. From this, different classification features are defined as energy ratios between the DWT components at different levels. These features are then used as inputs for an SVM-based multi-class classifier. Likewise, focusing on network layer measurements, Lu and Ghorbani [28] proposed a mechanism for network intrusion detection where normal daily traffic is modeled by wavelet approximation coefficients.

However, these types of transform-based approaches have not been applied for the analysis of cell-level indicators, typically being only used in an accessory role of the analysis (e.g., smoothing, periodicity detection) and not as the basis for the detection. Furthermore, the possibility of analyzing the different components resulting from a wavelet transform has not been developed. As an example, the work in [29] discussed the possibilities of using the fast Fourier transform (FFT) and wavelets for abnormal behavior detection. However, the paper adopted the use of Difference over Minimum (DoM) transformation, applied to smooth cellular metric values. The smoother versions are compared to the original ones to detect unexpected behaviors. Again, in the context of abnormal behavior detection, wavelets were also described as applicable for KPI smoothing in [30]. As mentioned, in both cases, the transforms are used for the smoothing of the metrics, missing the multiresolution information associated with the different trends obtained by transform-based analysis.

Similarly, Reference [6] applied FFT over already identified anomalies to establish their possible periodicity. The work in [31] followed a predictor-based approach for outage detection, using a transform related approach: Fourier series of residual errors were used to improve the estimation of user equipment signal measurements. With the objective of analyzing traffic burstiness, the authors of [32] applied the concept of self-similarity to the wavelet decomposition of network traces.

The identified related works, as well as recent surveys and the state-of-the-art analysis on the topic [7,11] further confirm the need to deepen the application of transform-based decomposition, and particularly wavelet-based, as the basis for novel techniques of multiresolution anomaly detection of cell-level metrics.

## 3. Methods

### 3.1. Wavelet Analysis

The cellular network, as composed of a huge number and variety of elements (base stations, sectors, terminals, and management and control elements), generates many different metrics. These consist of the time-series of the values of an indicator, KPIs, counter, alarm, trace, or any other parameter that is variable in time.

Transform-based decomposition is proposed as the basis for analyzing different behavioral trends in a metric. This is commonly achieved in integral-based transforms by the integral of the product between the analyzed time metric and a particular kernel function. The resulting output function depends on time (in particular, the interval of time where the integral is calculated) and an additional variable (e.g., scale/frequency), allowing distinguishing different components of the original metric from its different values.

Typical transforms include Fourier, such as short-time Fourier transform (STFT) [8,33], and wavelet [9] solutions. Wavelet transforms are particularly interesting in the analysis of non-periodic series, such as the sequential values of cellular network metrics. This type of transform returns information in both time and scale/frequency. For discrete transformations, the output of the transformation produces components at different levels that contain information about the original metric frequency-like behavior [34] corresponding to distinct frequency bands and different instants. These components are proposed as particularly suitable for the identification of degradations at different scales (temporal frequencies) in cellular network metrics, supporting improved detection performances.

The term wavelets refers to a particular set of functions used for transformation and multiresolution analysis of time-series [9]. These are wave-like oscillation functions constrained in time. For wavelet transforms, these functions are scaled at different levels, and their convolution with the input metric is calculated. This provides the information on the frequency/scale behavior of the input data.

**Figure 1:**
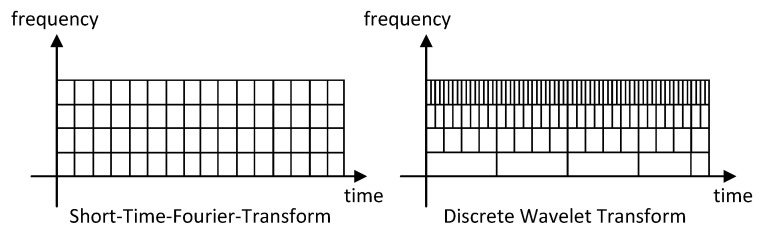
Time-frequency resolution of different transform-based decompositions.

Two key characteristics of the multiresolution transforms are their time and frequency resolutions. These refer respectively to the range of time and frequency covered for each value of the transform. Comparing the frequency and time resolution of wavelet transforms with other approaches, such as STFT (see Figure 1), wavelets provide good time resolution for fast changes in the decomposed metric while a good frequency resolution for slow changes. This is ideal for the analysis of metrics presenting daily and weekly persistent patterns together with quick non-periodic changes.

A special case of wavelet decomposition is the discrete wavelet transform (DWT), which consists of the application of the transform concept to a discretized version of the transform function considering only a particular set of scales.

Although the proposed system is not limited to the DWT solution, this introduces important advantages in comparison to other options. In particular, it allows the implementation of the decomposition (and further reconstruction) by means of discrete filter banks at reduced computational cost [35].

**Figure 2:**
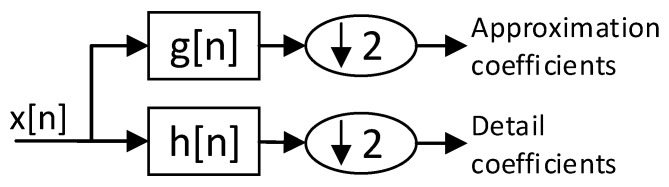
Block diagram of the proposed method.

The one level decomposition of an input set of values x[n] follows the scheme presented in Figure 2. In this way, x[n] is decomposed into low-frequency/large-scale coefficients (or approximation coefficients) and high-frequency coefficients (called detail coefficients).

The scheme is equivalent to, firstly, filtering the metric following the expression:

(1) $$cy_{high}\left[n\right]=\sum_{k=-\infty }^{\infty }x\left[k\right]h\left[2n-k\right],$$

$$cy_{low}\left[n\right]=\sum_{k=-\infty }^{\infty }x\left[k\right]g\left[2n-k\right],$$

where h[n] is the decomposition high-pass filter and g[n] is the low-pass filter forming a quadrature filter pair. The parameters $y_{high}$ and $y_{low}$ here represent the output of the high-pass and the low-pass filters, respectively.

Secondly, after the filtering, the samples are decimated, discarding half of them, as shown by the subsampling operator:

(2) (y↓2)[n]=y[2n]

**Figure 3:**
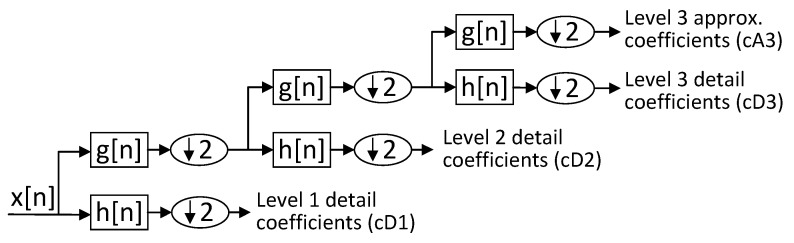
Bank filter for generating the coefficients of the discrete wavelet transform.

Figure 3 shows the reiterative application of this scheme by filter banks to obtain the coefficients at different levels.

**Figure 4:**
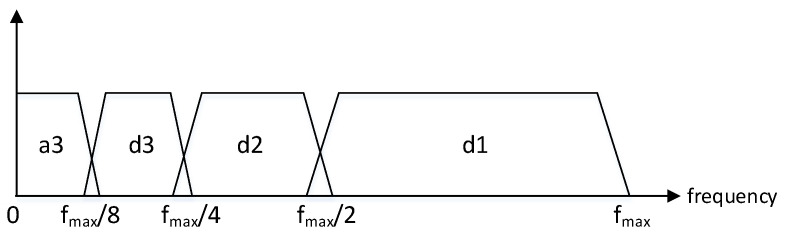
Discrete wavelet transform frequency ranges of the different coefficients.

Depending on the level, the coefficients would gather the behavior of the metric in different time scales/frequencies, as represented in Figure 4.

In this way, the coefficient $cD_{l}$, of each level *l*, encompasses a different frequency range of the metric spectrum:

(3) $$cD_{l}\to frequency\in \left[\frac{f_{max}}{2^{l}},\frac{f_{max}}{2^{l-1}}\right]$$

(4) $$cA_{l}\to frequency\in \left[0,\frac{f_{max}}{2^{l}}\right],$$

where $f_{max}$ refers to the maximum frequency of the metric. Considering discrete metrics and Nyquist sampling: $f_{max}=f_{s}/2=1/2T$, where $f_{s}$ is the sampling frequency and *T* is the measurement period of the metric.

Detection mechanisms could be able to directly work with the obtained coefficients. However, this would reduce the resolution of the analysis as the coefficients at each level provide only one value at each temporal period of their level. For the discrete case and the detailed coefficients, this leads to providing for each level *l* one transform value for each set of $\left[2^{l},2^{l+1}\right]$ samples of the metric. Instead, the system would be based by default on the reconstruction of the different components. These consist of the metric reconstructions at each level of the decomposition, where the components have the same length as the original metric.

The reconstruction is performed by following the inverted scheme of the one presented in Figure 3. Firstly, the coefficients are upsampled by a factor of two. Secondly, they are filtered by $g^{\prime }\left[n\right]$ (for $cA_{l}$) or $h^{\prime }\left[n\right]$ (for $cD_{l}$). The variables $g^{\prime }\left[n\right]$ and $h^{\prime }\left[n\right]$ are respectively the reconstruction low-pass filter and reconstruction high-pass filter. These, together with the decomposition filter h[n] and g[n], form a quadrature mirror filter system [36]. Thirdly, for coefficients with l>1, the result is upsampled and filtered by $g^{\prime }\left[n\right]$ l−1 times.

By this, the detail and approximate components at each level *l* are obtained, expressed as $d_{l}$ and $a_{l}$, respectively. Each component reproduces the metric behavior for the frequency band of their level, as defined in Equation (3). Considering the discrete nature of the metrics, these frequency bands can be translated into discrete temporal periods, Δη measured in the number of samples; in this way, $d_{l}$ period range $\Delta \eta_{d_{l}}\in \left[2^{l},2^{l+1}\right]$ samples and $a_{l}$ period range $\Delta \eta_{a_{l}}\in \left[2^{l+1},\infty \right]$ samples.

For the definition of the transformation filters (g[n], h[n], $g^{\prime }\left[n\right]$, and $h^{\prime }\left[n\right]$), different families of wavelets have been defined in the bibliography [37]. Each wavelet family is based on a set of mother wavelet functions Ψ, which share specific conditions and characteristic.

**Figure 5:**
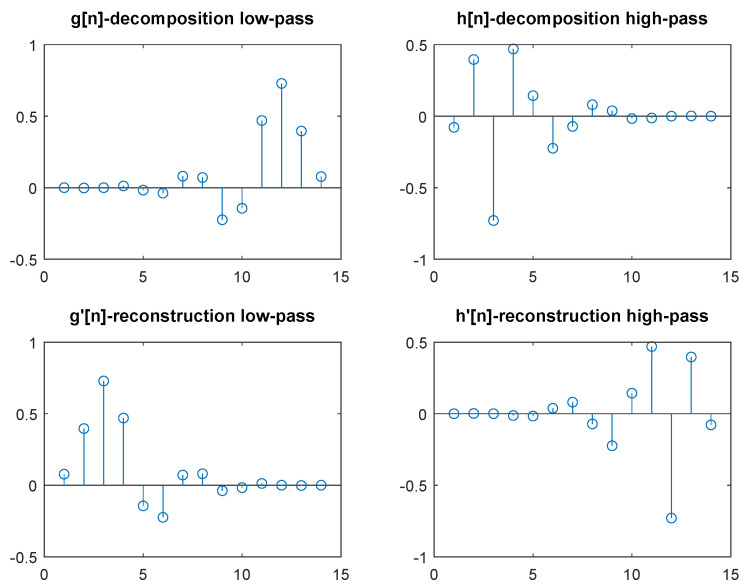
Transform filters of the wavelet type db7.

Each of these mother wavelets is the basis for the definition of a double pair of decomposition and reconstruction filters. For the proposed system, each set of four filters (g[n], h[n], $g^{\prime }\left[n\right]$, and $h^{\prime }\left[n\right]$) would be the wavelet type of decomposition. For example, the Daubechies family-based filters are commonly used [38]. As an example, the filters for the Daubechies wavelet of order seven (db7) are shown in Figure 5.

### 3.2. Proposed Framework

**Figure 6:**
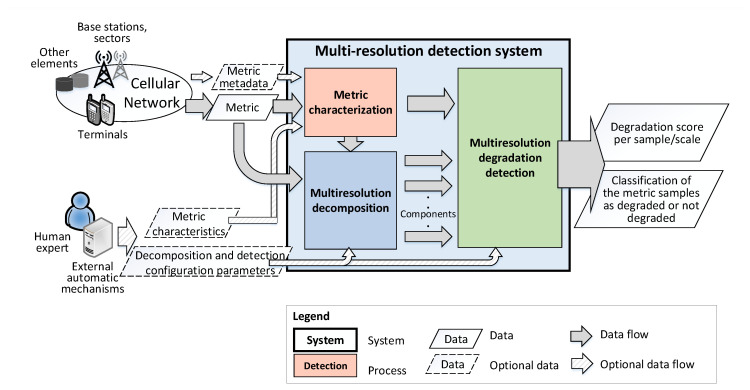
General system scheme in which the network metrics are analyzed for the detection of network degradations.

Having a cellular metric as the input, the proposed multiresolution detection system is based on the decomposition of these metrics in different steps. Figure 6 shows a high-level logical scheme of the system, distinguishing three main modules. Firstly, the metric characterization module is dedicated to properly identifying the main characteristics of the input metrics in case they are not present in the metric metadata, as described in Section 3. Secondly, the metric multiresolution decomposition block is dedicated to the application of the transform-based decomposition to the input metrics. Finally, the multiresolution degradation detection module oversees the processing of the resulting components coming from the decomposition to detect and characterize the degradations. From this process, the system returns two main outputs:

- A quantifiable degradation score indicating the level of the abnormality of the samples for each of the scales considered.
- The classification of the metric samples as degraded or not degraded and the scale where the degradation has been detected.

#### 3.2.1. Inputs

The proposed system can be applied to different metrics: counters, performance indicators, traces, or other measurement generated by base stations, user terminals, or other equipment of the cellular network. Here, a metric sequence (defined as a set of samples of the metric) is characterized and decomposed by the system in its time and scale components in order to identify anomalous behaviors. The components obtained from the decompositions allow the discovery of the degradations of different forms and duration. Given the association between the different levels of decomposition and their temporal ranges, it would also provide information about the temporal scale of the degraded behaviors in the metric sequence.

**Figure 7:**
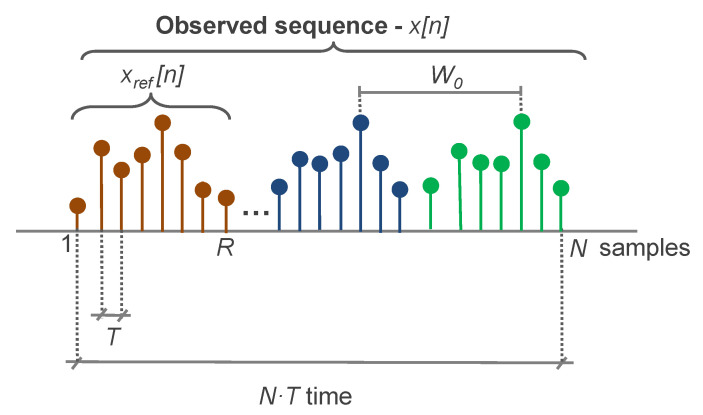
Metric scheme.

In this way, the first input of the system is a vector containing an observed sequence of a metric, x[n], composed of *N* samples (see Figure 7). Each sample corresponds to a measurement period of a *T* time duration, e.g., one hour. The sequence x[n] is therefore associated with a time interval of length N×T, e.g., 10 days, 48 h, etc. The system can be applied to the metrics in an online manner (i.e., to continuously received metrics) or offline (i.e., to previously recorded series).

Although x[n] can be used as the only input for the detection system, this means, without the use of the definition of specific thresholds, other inputs might be available, as shown in Figure 6. Human experts or external automatic mechanisms can directly provide data about the characteristics of the metric as optional inputs for the system. In addition, some information could be obtained from the metric metadata, which are stored in the operator’s databases, and they describe the characteristics of the metric (e.g., if it is a weekly or hourly indicator). Otherwise, they would be estimated from the analysis of the metric itself, as will be described in further sections.

Specifically, the required characteristics of the metrics include:

- The set of pseudoperiods of the metric, W, which includes different values of periodicity $W_{0},W_{1}\ldots$, where it is expected that the values of x[n] follow approximately the values of x[n−W]. The variable W depends on the nature of the monitored cellular network. For instance, the hourly metric typically follows a daily pattern that generally repeats each day. Additionally, the same dates in different weeks share common trends, associated with the varying distribution of workers’ activity through the week (e.g., Sundays have reduced activity). For the hourly metric, W={24,7×24,30×24}. For the set of pseudoperiods, the minor one would be identified as $W_{0}\in N$, e.g., $W_{0}=24$ for the hourly metric. The value $W_{0}$ can be derived from either the metric metadata or the automatic analysis of the metric (e.g., calculating the FFT of the available metric samples and obtaining the frequency component with higher energy).
- A reference sequence $x_{ref}\left[n\right]$ of *R* samples will be used as an indication of the normal behavior of the network or as a typical degradation pattern. For the best performance, $R>=W_{0}$. Ideally, *R* must be equal to or larger than any of the W periodicities. Hereafter, it is assumed that the signal $x_{ref}\left[n\right]$ consists of the initial *R* samples of x[n]. That is, if the first gathered samples of the metric are normal, the reference consists of those first *R* samples. If the first samples do not present a normal behavior, a set of *R* samples representing the normal state will be placed at the beginning of x[n].
  The definition of the reference sequence is not trivial, as it is difficult to establish a complete set of what can be considered normal behavior, and this normality will typically change in time and for different cells due to differences in network use, long-term variations, etc.
  Because of this, classical approaches typically imply additional inputs (e.g., expert knowledge) to define it. Conversely, the proposed system uses the complete input sequence as the reference; thus, R=N, $x\left[n\right]=x_{ref}\left[n\right]$. In this case, the system would be able to identify outliers at different scales. Outliers refer to those values that are outside of the usual range of the metric. These could not easily be identified from the original metric x[n] with classical techniques due to its periodic variabilities and trend behavior. However, the proposed system decomposition allows isolating each temporal trend, making it possible to identify the outliers at different scales.

In addition to the previous variables, certain parameters of the decomposition and detection process could be additionally configured by human experts or other external systems, as will be described in the following steps; for example, the type of transform, the level of decomposition, etc. In any case, automatic methods are defined to allow a fully independent execution of the system if such parameters are not introduced.

**Figure 8:**
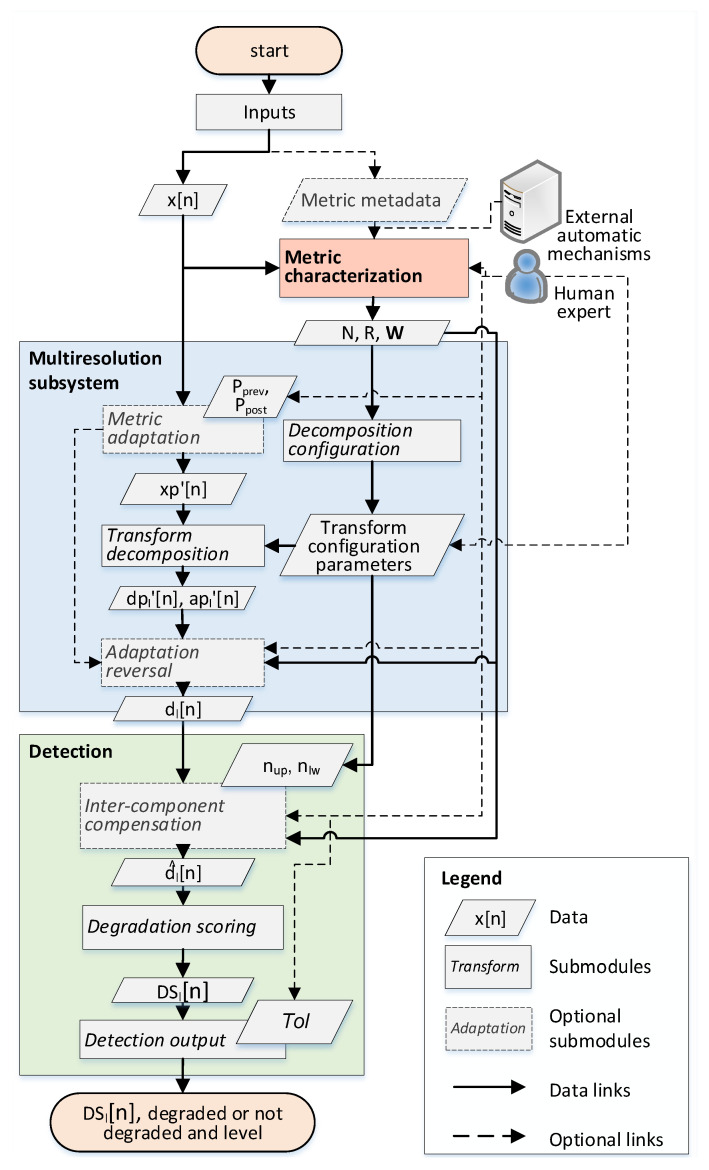
Steps of the multiresolution detection.

The steps and modules of the proposed system are detailed in Figure 8 and described in the subsections below.

#### 3.2.2. Metric Characterization

This step is dedicated to characterizing the input metric by gathering its main parameters as introduced in Section 3. In this way, *N*, the number of samples of the observed sequence, can be directly obtained by counting the number of samples of x[n]. The number of samples of the reference sequence, *R*, can be provided as an input if the labeled data, that is, a set of samples already identified as normal, are provided. If not provided, R=N is assumed for the unsupervised application of the posterior stages of the system.

Finally, the set of periodicities of the metric, ***W***, is calculated. For hourly indicators, W={24} can be assumed, and W={7} for daily ones. These periodicities would be typically automatically obtained from the metric metadata, such as its name and its associated information in the operator’s databases. Conversely, if this information is not available, the metric periodicities are estimated based on the analysis of the metric values by means of the fast Fourier transform of the metric, identifying the maximums in its spectrogram.

#### 3.2.3. Multiresolution Decomposition

This module performs the multiresolution decomposition of the original metric into different frequency/scale components. As mentioned, the key challenge of this module is to be able to process very heterogeneous input metrics in terms of the time scale (e.g., hourly, weekly), duration (e.g., from sets of data from a couple of days to several weeks/months), and present trends (e.g., daily/weekly/monthly patterns, long-term trends of increasing or decreasing use, etc.). To cope with this, in our proposed framework, the standard DWT (whose principles are described in Section 3.1) is supported by additional metric adaptation and transform configuration steps. In this way, the main submodules of this multiresolution decomposition module are the metric adaptation, decomposition configuration, transform decomposition, and adaptation reversal.

Metric adaptation is applied to improve the results of the decomposition by processing its original values in three aspects: normalization, shaping, and padding. Firstly, cellular metrics are typically defined in ranges (e.g., x[n]∈[0,∞) or x[n]∈[0,100] for percentage/ratio metrics). The disparate ranges between the different metrics can lead to computation and representation problems. To avoid these, the z-score normalized metric, $x^{\prime }\left[n\right]$, is calculated as:

(5) $$x^{\prime }\left[n\right]=\frac{x\left[n\right]-\mu_{x}}{\sigma_{x}},$$

where $\mu_{x}$ and $\sigma_{x}$ are, respectively, the mean and standard deviation of x[n]. $x^{\prime }\left[n\right]$ is then used as the input for the next steps.

Secondly, the quality of the transform-based decomposition methods is linked to the similarity of the analyzed metric in comparison to the used mother wavelet [39]. Therefore, its proper selection is deemed necessary. For this, both qualitative and quantitative mechanisms are considered useful. Moreover, shaping procedures to increment this similarity are therefore applied. An example is the periodical introduction of additional samples to increase the symmetry of the metric.

**Figure 9:**
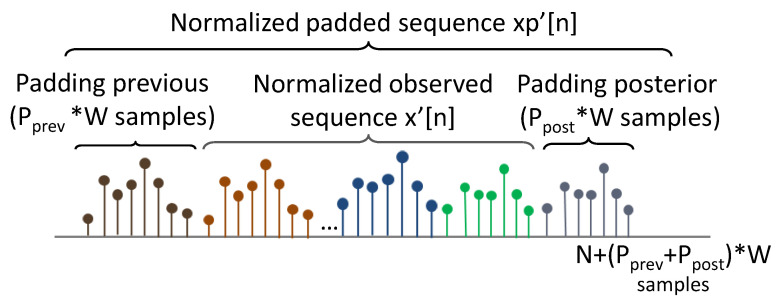
Padding process.

Thirdly, border effects in the transformation can limit the quality of the decomposition for the values located at the beginning and the end of the metric series. To avoid them, metrics are padded at the beginning (padding previous) and at the end (padding posterior), by concatenating a set of $W_{0}$ samples at both the beginning and at the end of the metric. These padding sequences are extracted from $x_{ref}\left[n\right]$, as shown in Figure 9. The length $N_{p}$ of the padded metric xp′[n] is therefore $N_{p}=N+\left(P_{prev}+P_{post}\right)\times W_{0}$. This step is especially important for series with a reduced number of samples (e.g., N<=48). For those, $P_{prev}=P_{post}=1$ can be established by default.

The decomposition configuration is executed to identify the parameters to be used in the posterior transformation as they would differ between different metrics. In particular, the parameters to be establish are:

- Transform type: By default, the discrete wavelet transform with db7 filters is chosen, being the one considered the most suitable for the analysis of cellular metrics (as described in Section 3.1). However, other discrete transforms (e.g., STFT) and kernel functions could be straightforwardly applied.
- Maximum level for detection ($l_{det}^{max}$): This refers to the maximum level of the components (provided by the decomposition) that will be considered for the detection subsystem.
  For the particular case of DWT, given the relation between the coefficient levels and the possible period of the component (see Equation (6)), the maximum level of the decomposition is estimated as:
  (6) $$l_{det}^{max}=log_{2}\left(floor\left(min\left(\frac{N_{p}}{F-1},\frac{R}{2\times minTREF}\right)\right)\right),$$
  which is the minimum between two limits rounded down to the nearest integer (floor function). The limit $N_{p}/\left(F-1\right)$ represents the maximum level of downsampling steps that can be applied to $xp^{\prime }\left[n\right]$ so that the output sequence has at least the same number of samples as the filter impulse response, denoted as *F* (where F−1 is the order of the filter). For example, F=14 for “db7”.
  The limit R/(2×minTREF) indicates the maximum decomposition level where the reference sequence would contain at least minTREF times the number of samples of its temporal period (see Equation (3)). This guarantees the statistical significance for the estimation of normal values during the detection phase (as at least minTREF periods of the higher-level component would be considered).
- Decomposition level (L): This is the highest level of the generated components for a discrete decomposition. It must be satisfied that $L>=l_{det}^{max}$. The decomposition *L* can be superior to $l_{det}^{max}$ for visualization reasons to provide further information of the metric behavior to a possible human operator or other systems. Furthermore, the components of levels higher than $l_{det}^{max}$ can be required for inter-component compensations, as is detailed in Section 3.2.4.

Transform-based decomposition is then implemented via the DWT of the metric adaptation block output, $xp^{\prime }\left[n\right]$, and considering the configuration parameters defined in the decomposition configuration. Here, the set of the different detail level components ${\mathrm{dp}}^{\prime }\left[n\right]$ represents the input for the next submodules. For each individual component ${dp}_{l}^{\prime }\left[n\right]$, *l* indicates its level. These can also be accompanied by the calculation of the approximate components ${ap}_{l}^{\prime }\left[n\right]$ (where *l* is their specific level) as described in Section 3.1. Typically, the approximate components are not required for the detection process. Each component reflects the metric behavior for a different frequency band, as shown in Figure 4 and defined by Equation (3) in Section 3.1. The frequency band of each component is expressed in terms of their temporal period range in the number of samples: ${dp}_{l}^{\prime }$ period $\in [2^{l},2^{l+1}]$ samples and ${ap}_{l}^{\prime }$ period $\in [2^{l+1},\infty ]$ samples.

Adaptation reversion procedures are then applied to revert the normalization/shaping/padding applied at the beginning of the multiresolution decomposition block. The resulting dp[n] components are unpadded, extracting the $P_{prev}\times W_{0}$ previous and $P_{post}\times W_{0}$ posterior samples to obtain the final components $\mathrm{dp}\left[n\right]=\{d_{l}\left[n\right],\forall l\in \left[1,L\right]\}$. Each of the resulting $d_{l}\left[n\right]$ components has a length of *N* samples, where each *n* sample of the original x[n] metric has a related value in each reconstructed component. In this way, a set of the samples of reference $d_{l}^{ref}\left[n\right]$ can be defined as the values that coincide with the samples of $x_{ref}\left[n\right]$.

#### 3.2.4. Multiresolution Degradation Detection

Once the different components have been extracted, the system implements the detection based on those components. Here, the proposed framework is not limited to any particular algorithm. However, complex mechanisms, such as neural networks, are computationally costly and usually imply strict learning requirements. Instead, an algorithm based on the identification of metric abnormal values (outliers) in the different components is proposed. A set of sequential steps is designed for the detection process: inter-component compensation, degradation scoring, and detection output.

Inter-component compensation is a block defined to cope with the fact that in the decomposition of long metric series (e.g., $N>7\times W_{0}$), it has been observed how some high-level components present relatively short “bursts” of high value, which minimize the changes corresponding to other levels/frequencies. To compensate this, for each particular component $d_{l}\left[n\right]$ and sample *n*, a “compensated” component $\hat{d_{l}}\left[n\right]$ of level *l* is calculated following Equation (7):

(7) $${\hat{d}}_{l}\left[n\right]=d_{l}\left[n\right]-\sum_{\rho =l+1}^{min(L,l+n_{up})}\left(\bar{d_{\rho }^{ref}\left(\mathrm{eq}\left[n\right]\right)}-d_{\rho }\left[n\right]\right)-\sum_{\varsigma =max(1,l-n_{lw})}^{l-1}\left(\bar{d_{\varsigma }^{ref}(\mathrm{eq}\left[n\right]}-d_{\varsigma }\left[n\right]\right),$$

where $\bar{d_{\rho }^{ref}\left(\mathrm{eq}\left[n\right]\right)})$ represents the average of the reference samples of a specific higher component $d_{\rho }$ (where ρ>l) in the equivalent samples of other periods, calculated as:

(8) $$\mathrm{eq}\left[n\right]=\{j\in \left[1,R\right]|mod\left(j,W_{0}\right)=mod\left(n,W_{0}\right)\wedge n\neq j\}$$

Equivalently, $\bar{d_{\varsigma }^{ref}(\mathrm{eq}\left[n\right]})$ represents the average of the reference samples of a lower component $d_{\varsigma }$, where ς<l. The variables $n_{up}$ and $n_{lw}$ indicate, respectively, the number of upper and lower levels used in the compensation. From the performed analyses, short series in terms of their periodicity (typically those with N<=7×W) do not require inter-component compensation. When applying this compensation process, the condition $L>=l_{det}^{max}+n_{up}$ shall be satisfied to have the required components. Therefore, by default, $L=l_{det}^{max}+n_{up}$.

Degradation scoring is then used to generate an anomaly indicator/score for each component. These scores represent the level of anomaly at each instant and decomposition level. The method proposed to calculate them is based on the statistical analysis of the components, supporting automatic applications of the framework in line with the statistical thresholding methods identified in Section 2 [3,15,17]. Here, to show the capabilities of the approach, the very simple technique based on categorizing as anomalous those samples that diverge from the mean by a number of standard deviations is used [40].

In this way, the mean and standard deviation of each reference component are calculated as follows:

(9) $$\mu \left({\hat{d}}_{l}^{\;ref}\right)=\frac{1}{R}\sum_{n=1}^{R}{\hat{d}}_{l}^{\;ref}\left[n\right],$$

(10) $$\sigma \left({\hat{d}}_{l}^{\;ref}\right)=\sqrt{\frac{1}{R}\sum_{n=1}^{R}{\left({\hat{d}}_{l}^{\;ref}\left[n\right]-\mu ({\hat{d}}_{l}^{\;ref}\right)}^{2}}$$

From these, for each level *l*, the lower $th_{lw}^{l}$ and upper $th_{up}^{l}$ normality thresholds can be calculated following the expressions:

(11) $$th_{lw}^{l}=\mu \left({\hat{d}}_{l}^{\;ref}\right)-Tol\times \sigma \left({\hat{d}}_{l}^{\;ref}\right)$$

(12) $$th_{up}^{l}=\mu \left({\hat{d}}_{l}^{\;ref}\right)+Tol\times \sigma \left({\hat{d}}_{l}^{\;ref}\right),$$

where the tolerance, Tol, tunes the strictness of the threshold, being the adopted solution by which values beyond three times the standard deviation away from the mean are considered degraded.

This configuration can also be used when no reference sequence is provided. In that case, the totality of the metric is used for the generation of the thresholds considering ${\hat{d}}_{l}^{\;ref}={\hat{d}}_{l}^{\;}$.

Based on these thresholds, a direct way to analyze the level of the degradation of a metric is by calculating the proposed degradation score per level, $DS_{l}\left[n\right]$, as:

(13) $$DS_{l}\left[n\right]=\frac{{\hat{d}}_{l}^{\;}\left[n\right]-\mu \left({\hat{d}}_{l}^{\;ref}\right)}{Tol\times \sigma ({\hat{d}}_{l}^{\;ref})};$$

hence, the generated adimensional normalized score is defined in the range (−∞,∞), where values in [−1,1] indicate no degradation (i.e., the thresholds are not crossed) and degradation otherwise.

Detection output is then the final procedure to identify, from the calculated $DS_{l}\left[n\right]$ values, the abnormal samples and their corresponding levels. The latter can be defined as those whose absolute value $|DS_{l}\left[n\right]|$ is above one. Moreover, knowing the level (*l*) of the component crossing the threshold provides information about its frequency range characteristics. A degradation in level *l* is a result of an abnormal behavior during one semiperiod of the component, $\nu_{l}$. Given the expression for the period range of each component in Equation (3), the semiperiod range is defined as:

(14) $$\nu_{l}\in \left[2^{l},2^{l+1}\right]/2=\left[2^{l-1},2^{l}\right]\;samples,$$

indicating the time-behavior of the metric that led to the abnormal value. For example, a degraded value detected in the component l=3 of a daily metric would be associated with a degraded trend in the semiperiod range of 4 to 8 days, which would likely be caused by a week of abnormal behavior.

## 4. Evaluation

To assess the contribution and capabilities of the proposed system, a set of baseline approaches was selected. These are described below, indicating the acronym that will be used to identify them in the posterior evaluation. Moreover, how the level of degradation of each sample is valued for these methods is defined. Whereas the original references did not provide a standardized degradation score with values in [−1,1] indicating no degradation, this kind of score will be adopted for each of them. In this way, the details of three implemented baseline mechanisms are described below:

- Metric Statistical Threshold (MST): The level of anomaly of each sample metric x[n] is directly measured by how far it is from its mean values, also considering a tolerance associated with its standard deviation (three times) [40]. The calculation of the degradation score is here equal to the one defined in Equation (13), but calculated directly from the metric instead of the components.
  (15) $$DS_{MST}\left[n\right]=\frac{x[n]-\mu (x[n])}{Tol\times \sigma (x[n])},$$
  where Tol=3 and the statistics are calculated for the complete input metric x[n].
- Forward Linear Predictor (FLP): Following a similar scheme to the one in [24], a method based on the forward linear predictor of the 10th order is implemented, representing the predictor-based approaches discussed in Section 2. The prediction absolute error $|e\left[n\right]|=|x\left[n\right]-x_{pred}\left[n\right]|$ for each sample is the value used in this type of approach to detect anomalies. The original work [24] established some formulation in order to distinguish between metrics where the sign of the degradation is relevant, as well as assuming a zero mean for the error. However, a degradation score as the one for MST is in terms of the normalized error, this being fully consistent with the original approach:
  (16) $$DS_{FLP}\left[n\right]=\frac{e[n]-\mu (|e[n]|)}{Tol\times \sigma (|e[n]|)},$$
  where again, Tol=3, consistent with the values in [24], and the error is calculated for the complete input metric x[n] (no reference period).
- Degraded Patterns’ Correlation (DPC): The approach in [22], referenced in Section 2, works by establishing a certain pseudoperiod or set of pseudoperiods of x[n] as reference, e.g., a period of 24 h. Degraded patterns are then generated by adding a negative or positive synthetic pattern (e.g., a positive-sign impulse or negative-sign impulse) to the reference sequence. This is done for all possible shifts of the synthetic pattern inside the reference sequence (e.g., the reference with a positive-sign impulse at *n* = 0, 1, 2, …24). The Pearson coefficient r1 is calculated for all these possible degraded patterns and each pseudoperiod of the remaining of x[n] under analysis. High values of this correlation r1 might indicate an anomaly of x[n], whereas the level of correlation r2 between the original reference set (without added patterns) and the pseudoperiod under analysis is also taken into account for the detection decision. Hence, fully complying with the original definitions in [22], the associated degradation score (with values outside [−1,1] to be considered degraded) is defined as:
  (17) $$DS_{DPC}\left[n\right]=q\times \left(r1-r2\right)+r1,$$
  with q=3. For this technique, the score will be calculated for both a positive impulse degradation pattern (labeled as DPC+) and a negative one (DPC−) [22]. This mechanism requires a reference sequence, which is one of its main shortcomings in respect to our proposed method.

To evaluate the proposed system in comparison with these baseline methods, a dataset from a real urban macrocell LTE network (covering a large city) was used following a similar approach to the one followed in [22]. From this dataset, a set of degraded metrics is identified that follow common faulty behaviors, which are difficult to identify by classical detection methods. First is the two hourly metric, with a pronounced daily pattern. The first one presents an impulse-like degradation consisting of a value lower than the one expected. The second one shows a degradation with a higher value than the normal. Thirdly, a daily metric presenting multiple types of degradations through multiple weeks is analyzed.

The detailed characteristics of these cases and their anomalies are provided at the beginning of the next subsections. In their analysis, all the parameters use the automatic settings indicated in the description of the system, comparing its results with the ones obtained by the baseline mechanisms in terms of the capacity to detect the existing anomalies and the closeness of the degradation scores to generate possible false positives.

### 4.1. Hourly Metric, Down-Degradation

**Figure 10:**
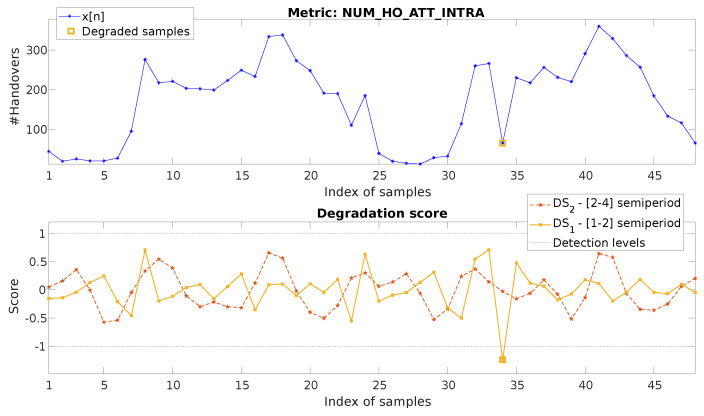
Two-day data of the hourly metric NUM_HO_ATT_INTRA and its decomposition and detection process following the proposed system.

Firstly, as shown in Figure 10-top, data from an hourly metric, the number (#) of intra-handover attempts (NUM_HO_ATT_INTRA), is analyzed. As can be observed, this metric is degraded during 1 h in the 34th sample, showing a low number of attempts in comparison with the previous day and considering the values shown in the hours before and after the affected hour.

For the detection, no reference period is selected, which means that the totality of the input metric is used as the reference: N=R=48. The decomposition is automatically configured with the parameters: W=24, $P_{prev}=P_{post}=1$, $n_{up}=0$, $n_{lw}=0$. This gives $l_{det}^{max}=2$, and no inter-component compensation is applied given that N<=7×24. The degraded score values ($DS_{l}\left[n\right]$) of each component are shown in Figure 10, bottom. The legend of the figure indicates the component from which each degradation score was obtained. The associated semiperiod range for each component level is shown between brackets.

Based on these values, the system detects n=34 as the point where the degradation score $DS_{1}$ crosses the detection level. The degraded component is therefore at Level 1, $d_{1}$. This indicates its association with a network degradation of duration in its semiperiod range. Following Equation (14), this means $\nu_{l}\in \left[1,2\right]$ samples (as indicated in the figure legend), which coincides with the faulty behavior shown by the metric. The detection output is also represented in Figure 10, top and bottom, where the larger orange square indicates the period detected as degraded and its association with $DS_{1}$.

**Figure 11:**
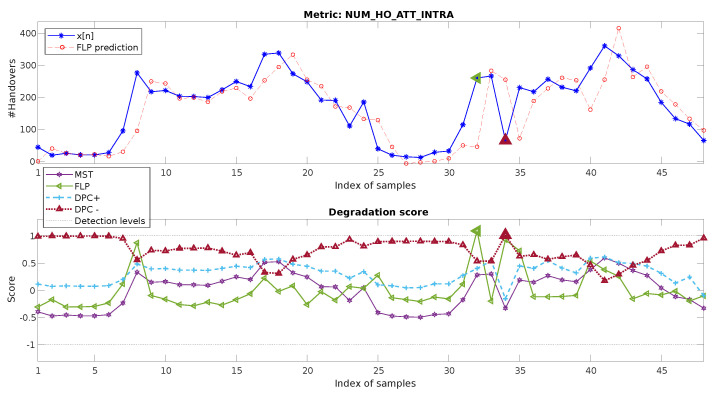
Two-day data of the hourly metric NUM_HO_ATT_INTRA, its prediction using the baseline linear filter (**top**), and its absolute prediction error (**bottom**). FLP, Forward Linear Predictor; MST, Metric Statistical Threshold; DPC, Degraded Patterns’ Correlation.

In order to compare these results with the proposed baseline mechanisms, the same dataset is processed with the MST, FLP, and DPC+/DPC− approaches as described at the beginning of the section. Both FLP and DPC use the first 24 h as training, while MST considers the complete x[n] for the statistics calculation. The results are represented in Figure 11. The top image shows the original metric and the one predicted for FLP. The bottom figure represents the degradation scores calculated for each of the baseline approaches.

For MST, it can be seen how, as the degraded value on n=34 keeps between the margin of the distribution of x[n], it is far from being detectable. This was expected as MST does not incorporate the temporal dimension in any manner. FLP is also not able to identify the failure: multiple samples provide a similarly high prediction error, making it not possible to define a threshold allowing the identification only of the unique degradation on n=34. In fact, with the considered score, a false positive is detected for n=32, as the predicted starting increase of traffic following the daily pattern has a delay of one hour, creating a peak in the error.

For DPC+, unsurprisingly, the mechanism is able to detect the n=32 degradation, as shown for the same metric in [22] and considering that the anomaly fits perfectly with a negative sign impulse. However, in order to achieve this, a reference set has to be established as normal (in this case, the first 24 h), information that is not always known. Additionally, the synthetic impulse patterns have to be defined. Moreover, it can be observed how the score values are very close to the threshold for many other samples, making it prone to possible false positives.

### 4.2. Hourly Metric, Up-Degradation

**Figure 12:**
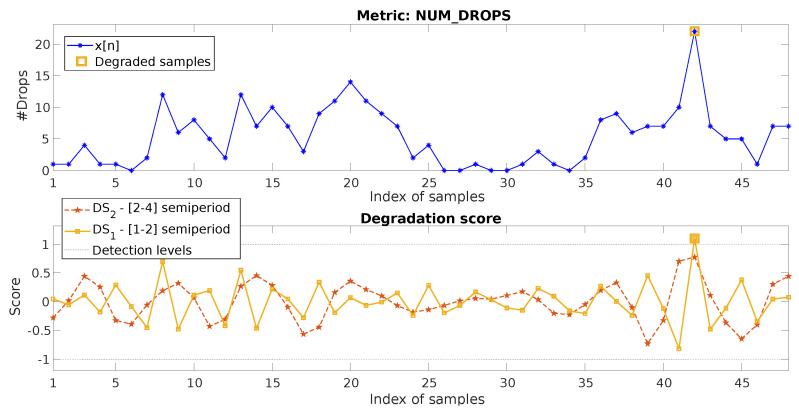
Two-day data of the hourly metric NUM_DROPS and its decomposition and detection process.

The data for this example are represented in Figure 12, top, which shows the hourly metric NUM_DROPS (number of dropped calls). As observed, the time variability of this metric is high, showing a degradation peak (increase in the number of drops) of a magnitude that is considerably greater than the values in the previous day and the adjacent samples.

For this case, as shown in Figure 12, bottom, the degradation score values ($DS_{l}\left[n\right]$) for n∈[25,48] show values higher than one only for n=42. The method allows identifying the degradation for n=42. Again, this occurs in the component $d_{1}$, as it is associated with one unique sample.

**Figure 13:**
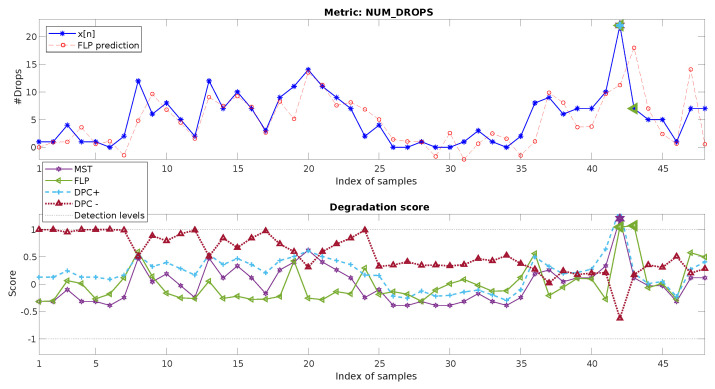
Two-day data of the hourly metric NUM_DROPS, its prediction using the baseline linear filter (**top**), and its absolute prediction error (**bottom**).

Figure 13 presents the results for the baseline methods, where this case is somehow easier than the previous one to address for them. However, their performance is still far from the one achieved with our proposed multiresolution mechanism. MST is able to detect the anomaly for n=42 given that the value goes quite far (more than three standard deviations) from the mean of x[n]. FLP and DPC use again the first 24 h as training. For FLP, although in this case, the score allows detecting the degradation, this would lead to a false positive for n=43, wh8ch presents an even higher prediction error.

For DPC+, as the failure fits quite well with a positive sign impulse degradation pattern, the anomaly is identified. However, again, knowledge of the reference part of the metric, as well as of the type of possible degradation must be available for the process. Moreover, it can be observed how many values of the score are quite close to the threshold, showing that false positives could easily appear in the analysis.

### 4.3. Weekly Metric, Multiple-Degradations

**Figure 14:**
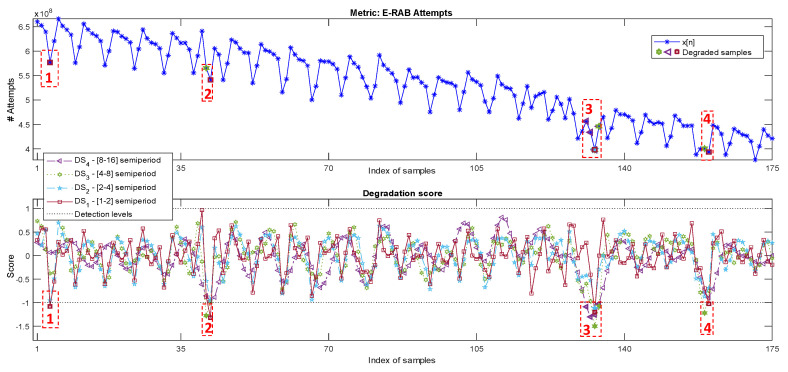
The E-RABattempts daily metric and its decomposition and detection process.

This last case presents a long and complex metric set. Here, the daily counter E-UTRAN Radio Access Bearer (E-RAB) attempts (number of attempts of the user equipment to connect to the network) of a cell are analyzed for 175 samples (25 weeks, one sample per day). As shown in Figure 14, top, this metric consists of a large sequence of values showing a strong long-term decreasing trend. It also shows a periodic behavior with a period of seven samples, associated with the weekly use pattern variations. These conditions would make impractical most of the common approaches of correlation or fixed threshold presented in Section 1.

The metric presents degradations of different characteristics and duration, as indicated by the numbered dashed red squares:

- Degradation 1: n∈[4,10] shows a typical one-week pattern, but of a duration of six samples, instead of seven.
- Degradation 2: There are anomalous low metric values in n=41 and n=42 (part of the week pattern of n∈[38,45]).
- Degradation 3: A sequence of more than one week n∈[128,136] has an overall out-of-trend reduction of the metric values. Furthermore, n=132 and n=133 show values breaking the normal weekly pattern.
- Degradation 4: n=159 and n=160 present degraded values.

For this case, the configuration parameters are those assigned automatically considering it as a long metric with no predefined reference set: N=R=175, W=7, $P_{prev}=P_{post}=0$, $n_{up}=2$, $n_{lw}=1$.

The $DS_{l}\left[n\right]$ values are shown in Figure 14, bottom. The dashed red squares encompass the points of detected degradation, also indicated by wider edge line markers. It is observed how, as expected, Degradation 1 is detected based on the values of the degradation score $DS_{1}$, marking the lack of one day from the weekly pattern. Degradations 2 and 4 are correlated with $DS_{1}$ and $DS_{3}$ crossing the detection threshold, showing a degraded character at both weekly and daily levels.

Degradation 3 is detected by $DS_{4}$ in the segment n∈[131,134], related to the generally low values of the period n∈[128,136], a complete week. Furthermore, it is observed that, within this abnormal interval, a superimposed degradation by $DS_{1}$ can be found in n=133, which responds to a value that breaks the general degraded weekly pattern of the period even further.

**Figure 15:**
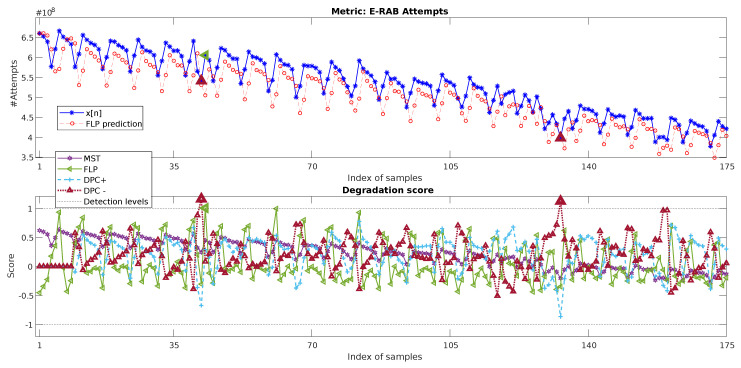
Two-day data of the daily metric E-RAB attempts, its prediction using the baseline linear filter (**top**), and its absolute prediction error (**bottom**).

The results of the baseline approaches can be observed in Figure 15. MST is not able to detect any anomaly again since the mechanism does not consider the evolution in time and the degradations being in the range of the three standard deviations limit. This is an especially complex case for this kind of approach as the non-stationarity of the metric due to its long-term trend increases the tolerance range defined considering a normal standard deviation enormously.

For FLP and DPC, considering the large number of samples of the dataset, three normal weeks are selected for training (n∈[10,31]). Once trained, FLP does not provide a proper detection of the degradations as the prediction error presents a repetitive pattern very similar for all weeks, with or without the presence of anomalies. Just one sample associated with Degradation 2 (see Figure 14) is detected. DPC- only detects an anomalous sample for Degradation 2 and another one for Degradation 3. Its score is also close to the detection of two samples associated with Degradation 4. However, these techniques do not provide any additional information on the nature of the anomaly behavior. They also require a proper selection of the normal reference period. In particular, for this case, the reference cannot be obtained just from the beginning of the time-series due to the presence of Degradation 1.

The analyzed cases show the capabilities of the proposed approach to provide the detection of network anomalies, as well as additional information on the characteristics of the degradation in a fully automated manner and for the heterogeneous metrics granularities, length, behavior, and degradation patterns. These advantages are prominent in comparison with the tested baseline methods, which required additional human-based configuration without providing a proper detection of the anomalies and being more prone to false positives.

## 5. Conclusions and Outlook

Covering the lack of previous works on the topic, the present work proposes an application of transform-based decomposition for the automatic detection of cellular network failures. A complete framework was developed in order to apply the decomposition in an automatic manner for heterogeneous cell-level metrics. As hypothesized, the use of transform-based decompositions, supported by the framework modules dedicated to the transform configuration and metric characterization and adaptation, allows applying simplified detection methods with improved detection performance for multiple metrics.

In this way, the provided evaluation shows the capabilities of our approach to overcome the presence of periodic patterns such as weekly or daily ones and long-term trends identifying the degradations of different shape and duration without the need for establishing specific reference periods or degradation patterns.

Future works are expected to deepen the application of transform-based decomposition for cellular management. Here, additional mechanisms for anomaly detection classification are expected to be also benefited by the application of the proposed framework. Moreover, root cause analysis applications are deemed promising, where the diagnosis systems are expected to be benefited by the transformation of the input metrics.

## 6. Patents

The developments of the present work have been partially subject to a patent application [12].

## Figures and Tables

**Table 1:** Summary of key related works on anomaly detection.

Anomaly Detection and Thresholding in Cellular Networks			
Category	Techniques	Summary	Ref.
Human thresholding	State machine	Degradation interval identification based on the crossing of manually defined thresholds.	[13]
	Entropy minimizationDiscretization	Calculation of numerical thresholds from labeled metric samples.	[14]
Statistical thresholding	Deviation from average, probability distributioncomparison, discretization	Thresholds based on statistics coming from normal samples or the average of the observed samples.	[3,15,16,17]
ML classifiers	Naive Bayes classifier, kNN, SVM, etc.	Automatic training based on labeled data (normal and degraded). Often, classification is based on the metric values without considering the time variable.	[16,18]
Patterns’ comparison	Correlation, clustering	Comparison of the observed time-series with normal/healthy patterns from the past or neighbor cells, synthetic degraded patterns, or contextual sources.	[19,20,21,22,23]
Predictor-based	ARIMA, forward linearpredictors, LSTM, etc.	The error between the predicted metric and the observed one is used as a degradation score.	[18,24,25]

**Table 2:** Summary of related works on transform-based analysis. DoM, Difference over Minimum.

Transform-Based Applications		
Field of Application	Transform	Ref.
Phonocardiogram signals	STFT, CWT	[10]
Network traffic anomaly detection	DWT	[26]
Packet length anomaly detection (network layer traces)	DWT	[27]
Network intrusion detection	DWT	[28]
Cellular metric smoothing	DoM	[29]
Cellular metric smoothing	Wavelet	[30]
Degradation periodicity identification	FFT	[6]
UE-level measurement prediction	Fourier series	[31]
Traffic burstiness identification	Wavelet	[32]
